# Athletic Identity in Youth Athletes: A Systematic Review of the Literature

**DOI:** 10.3390/ijerph18147331

**Published:** 2021-07-08

**Authors:** Bianca R. Edison, Melissa A. Christino, Katherine H. Rizzone

**Affiliations:** 1 Children’s Hospital Los Angeles, Department of Surgery, Division of Orthopaedics, University of Southern California, Los Angeles, CA 90027, USA; 2 Boston Children’s Hospital, Division of Sports Medicine, Department of Orthopaedics, Harvard Medical School, Boston, MA 02115, USA; Melissa.Christino@childrens.harvard.edu; 3 Orthopaedics and Pediatrics, University of Rochester Medical Center, Rochester, NY 14642, USA; Katherine_Rizzone@URMC.Rochester.edu

**Keywords:** athlete identity, adolescent athlete, athlete identity measurement scale

## Abstract

Athletic identity (AI), the degree of personal connection to sport, is well-described in adult research; however, this social trait has been less studied in younger age groups. This systematic review describes epidemiological characteristics of AI in youth athletes. PubMed, Embase and PsycInfo were searched to identify AI studies involving quantitative athlete identity outcomes and cohorts 22 years and younger. The search strategy was developed for each database using the Boolean method. PRISMA guidelines and the Appraisal Tool for Cross-Sectional Studies (AXIS) were utilized. Ten out of ninety-one studies met inclusion criteria. AI scores differed by race/ethnicity. Two studies found increased AI during adolescence compared to later in ones’ training. Mental health-focused studies revealed higher AI levels protect against burnout, but in injured athletes, increased depression risk. Transitioning to a higher level of play during adolescence can correlate with stronger senses of AI. Further research should explore the concept of athletic identity saliency as one moves through an athletic career or training program and how thoughts of perceived success, professional progression, recruitment prospect or injury affect levels of athletic identity.

## 1. Introduction

The psychologist William James considered the concept of “self” as a primary element of human thoughts, feelings and actions [1]. Several theories about identity have been proposed: personal versus social, singular versus multiple. In 1968, Erickson postulated that identity is primarily an unconscious and ever-changing sense of who one is, both as an individual and as a member of a particular group or society. One may self-identify simultaneously as a student, teacher, musician, spouse or parent. Erickson also declared that one’s identity cannot exist out of isolation from one’s culture or environment; identity development is inextricably linked to the core of an individual as well as the core of one’s communal culture [2]. Social identity theory proposes that identities are manifestations of a person’s identification with a particular group or social category, such as a sport team. When an individual perceives that a group may offer certain advantages or benefits, that particular membership is valued. This organically leads to displaying preference to that group as identity affirmation and inclusion is sought. Within identity theory, some argue that one’s social setting can assign an individual a role, but ultimately internalization of that role rests on the acceptance of the individual. Identity can exist as role-based self-conceptions with perceived expectations related to the performance of a social role [3], such as an athlete accepting a role attributed from a coach, team or audience. Contrarily, other athletes may reject externally applied expectations set forth, such as Charles Barkley’s rejection of a publicly assumed voluntarism to be a “role model” as a celebrity athlete [4]. Moreover, social identity theory proposes that identities can become fluid; categories precede the individual and identity recognition rests on negotiating in and out of group positions within one’s social environment, potentially shifting during one’s life or career. For example, does an athlete’s identity shift within their participation on a particular team, level of competition or when considering retirement from a sport?

Sport psychology research on athletic identity began in earnest in the 1990s and Britton Brewer was one of the first researchers to propose the concept of an athletic identity and systematically study this area of interest. Athletic identity refers to the degree of strength and exclusivity to which a person identifies with the athlete role or the degree to which one devotes special attention to sport relative to other engagements or activities in life [5]. Prior to the concept of athlete as a category for identity, research focused on personality types that athletes possessed to enable success and compare those to non-athletes [6]. Other early researchers included Eldon Snyder and Jay Coakely, with Snyder proposing a multiple role theory in which adolescents assume roles to include that of student and of athlete and that participation in sports can enhance individual outcomes, such as academic performance [7]. Athlete identity can exist on a large spectrum, as a small part of who someone is all the way up to a large encompassing part of his or her life [8]. This component of self-concept can be affected by the experiences, relationships and involvement of the athlete in sport activities.

Under the auspice that identity can be both psychologically and socially based, the greater emphasis one ascribes to their athlete role, the more likely one’s self esteem, motivation and outlook may be influenced by perceptions of athletic competence, performance and achievements. More recently, Brewer’s research shifted to more specific issues such as anxiety, mental toughness, optimism and the concept of lasting or fading athletic identity in the setting of ceasing participation (retirement).

Brewer, Van Raalte and Linder’s research included some of the first comprehensive efforts to conceptualize and systematically study athletic identity [5]. These researchers suggested that athletic identity functions as a cognitive structure and as a social role, stemming both from individual emotional connection and from feedback from others, such as teammates, coaches, parents and spectators. As a result of that research, a standard psychometric instrument was developed for the measurement of athletic identity. The Athlete Identity Measurement Scale (AIMS) has been validated and applied to elite athletes, recreational athletes and non-athlete populations. This questionnaire originally consisted of 10 items where possible responses range on a 7-point Likert scale from “strongly agree” to “strongly disagree,” with higher numerical scores equating to a greater link of self to sport. Athletic identity was initially conceptualized as a unidimensional construct but eventually transformed to be re-conceptualized into a seven-item questionnaire that consists of three dimensions: social identity, exclusivity and negative affectivity [9]. Social identity represents the extent to which individuals view themselves as occupying an athlete role, exclusivity represents the extent to which an individual’s identity and self-worth are determined solely by the performance in the corresponding athlete role and negative affectivity represents the extent to which an individual experiences negative affect in response to undesirable outcomes within an athletic role and space [10]. The AIMS has been validated using youth student samples and has been shown to have robust test-retest reliability and internal consistency ([9,11], Appendix B).

Other scales that measure athlete identity include Anderson’s Athletic Identity Questionnaire (AIQ) for Adolescents. This validated 21-item measure assesses four dimensions of self-knowledge for the concept of athletic identity to include Appearance, the Importance of exercise/sport/physical activity to the self, perceived Competence and Encouragement from others in participating in activities ([12], Appendix C). While Brewer’s AIMS measurement tool targets athletes and an athletic role in social environments, Anderson’s AIQ arose from an interest in evaluating general populations and the process by which the label “athletic” is attributed, evaluated and maintained [9,12].

Athletic identity has been shown to positively contribute to one’s emotional connection to sport and have beneficial effects on involvement in physical activity and athletic performance [13]. Research has found a positive correlation between athletic identity and commitment to training and sport goal orientation [14]. Higher performance outcomes and levels of enjoyment of sports have been reported by athletes with stronger athlete identity as compared to their peers who are less invested in an athlete role [9,13]. Brewer’s research also found that those who favor an athletic component of self-concept are more likely to be physically active than those who do not [9,11]. Conversely, other studies have shown that individuals with strong athletic identities may suffer detrimental effects of that association. Steinfeldt linked higher levels of athletic identity to lower tendencies to seek help and to higher levels of gender role conflict [15]. An additional negative impact of robust athletic identity includes use of performance-enhancing strategies. Athletes with significant athletic role identification were found to display behaviors that stem from over-conformity to the sport or athlete ethos, prompting one to push boundaries to keep that identity or continue participation in a particular sport, such as using anabolic steroids or other performance enhancing substances, overtraining, playing while injured or adopting disordered eating patterns [8,16]. The drive to play, compete, strengthen oneself and win can influence these athletes to engage in the aforementioned behaviors, despite knowing risks. In addition, injury or personal setbacks can negatively affect an athlete’s involvement, self-esteem and motivation, especially when athletic identity is assumed out of neglect of other social roles [17]. Those with an exclusive or strong athletic identity may find exceptional emotional difficulty adjusting to ceasing sport participation or retirement [9,11,18]. Athletes’ perceived success or lack thereof can also affect levels of athletic identity. Brewer et al. [19] revealed that over the course of a sports season, athletes’ AIMS scores can fluctuate, depending on performance, thereby pointing to the effects one’s valuation of achievements or failures can have on self-esteem and identity.

Recent trends in youth athletics have led to an increased emphasis on sport. The youth sport environment has evolved to mirror societal demands for increased competition and a more elite environment. As such, one of the trends to follow suit has been the rise in both prevalence and socially-driven promotion of youth sport specialization. Ericsson, Kramp and Tesch-Romer’s study of music and expert performance contributed to a resurgence of interest related to high volume practice in sport to the development of expertise [20]. Numerous studies have set models and frameworks to study and understand youth sport specialization with earlier research using a binary definition (participation in one single sport versus multiple sports) as a basis to group subjects and help guide the structuring of youth sport participation pathways [21,22]. Jayanthi et al. first proposed a degree of specialization model that incorporates other criteria such as year-round training and playing in one main sport at the exclusion of all others. This degree model serves to evaluate an athlete on a continuum [23]. The recent surge of youth sport specialization is thought to be a result of the idea that a large quantity of intense sport-specific training and early specialization provides a direct path toward adult elite sport achievement [24]. Incorporating Brewer’s theory of athlete identity and participation, athlete identity levels may be following this trend of higher specialization but could also coincide with the detrimental effects of high specialization rates in youth, to include increased incidence in overuse injuries in pediatric athletes, higher commercialism of sport [25] and higher rates of burnout/sport dropout [26]. It is unclear how athletic identity relates to sports participation to exclusion of other sports, recovery from injury and the sporting culture in the pediatric population. The objective of this study was to systematically review the literature to investigate and describe the epidemiological characteristics of athletic identity in the youth athlete population.

## 2. Materials and Methods

This review was registered at PROSPERO International prospective register for systematic reviews (#168594) and can be found at: https://www.crd.york.ac.uk/prospero/display_record.php?RecordID=168594, accessed on 15 April 2021.

### 2.1. Search Strategy

**Figure 1:**
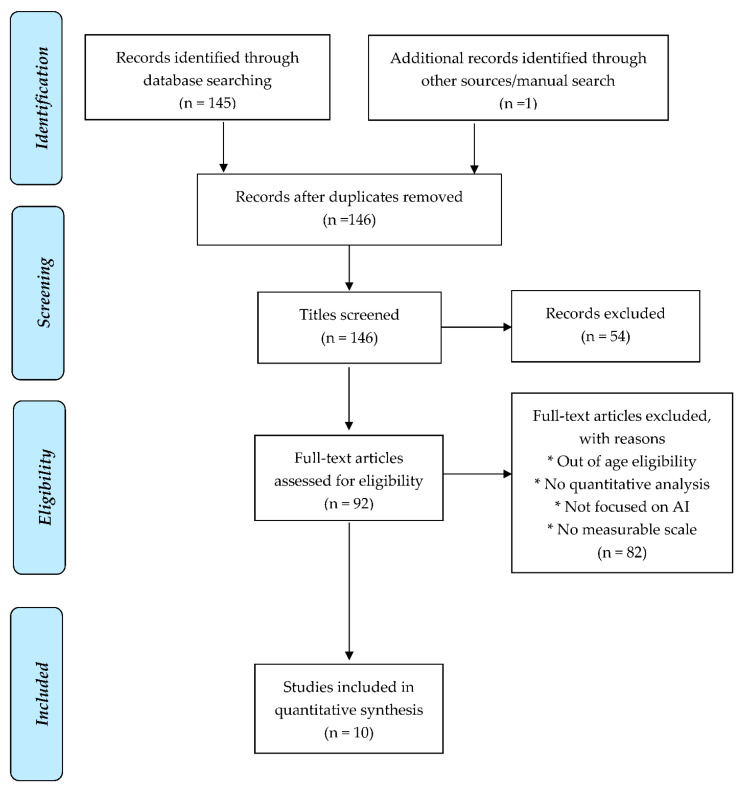
Title Screen, Abstract Screen, Full-Text Screens. From: Moher D, Liberati A, Tetzlaff J, Altman DG, The PRISMA Group (2009). Preferred Reporting Items for Systematic Reviews and Meta Analyses: The PRISMA Statement. *PLoS Med.* 6, e1000097. doi:10.1371/journal.pmed1000097. For more information, visit www.prisma-statement.org, accessed on 10 October 2019.

This review was conducted according to the Preferred Reporting Items for Systematic Reviews and Meta-Analyses Group (PRISMA) guidelines. 1. PubMed, Embase and PsycInfo databases were utilized in the literature search by an experienced librarian. Year 1945 to present day were included and limited to English-language publications. The search strategy was developed for each database using the Boolean method and used a combination of MeSH terms and free text, which was performed by an evidence-based librarian in October 2019. An example of the full search strategy is reported in Appendix A. Study eligibility was assessed independently by the three investigators by screening the titles, keywords and abstracts of each study. If a study appeared to meet eligibility criteria or if there was a question of its relevance, full texts were screened and assessed. Reference lists of retrieved studies were also hand searched for further relevant articles (Figure 1). Endnote X7 reference management software (Thompson Reuters, CA, USA) was used to ensure there were no duplicates.

### 2.2. Eligibility Criteria

Studies of inquiry were deemed eligible if the following inclusion criteria were met: (1): the original study was published in an English-language, peer-reviewed journal; (2) participants were aged 22 years and younger; (3) the study included quantitative outcome measures relating to athletic identity. Qualitative studies were excluded. Relevant information was extracted by the three investigators and checked for accuracy and omissions by a second author. Discrepancies were resolved by discussion and consensus. For each study, the following data was extracted: study design, participants’ characteristics and outcomes of interest. To avoid duplicating the inclusion of studies that involved the same cohort across multiple publications, the research team discussed and selected the outcome measures that were unique data points.

### 2.3. Data Extraction and Risk of Bias

Data extraction included study design, participant characteristics and athlete identity scale measures. Data were extracted by all three authors. Data were extracted through use of a standardized form. Differences in study selection were resolved by discussion and consensus. Methodological quality and risk of bias was independently assessed by all three authors using the Appraisal tool for Cross-Sectional Studies (AXIS). This 20 component scale rates studies for five different areas of construct: introduction, methods, results, discussion and other [27]. Each component was rated on a three-point scale as strong/yes, moderate/not reported or weak/no and a stoplight grading system was utilized by the study team. Green for strong/yes, yellow for moderate/not specifically reported and red for weak/no. Any disagreements were discussed with all reviewers until a consensus was reached.

### 2.4. Data Analysis

Following data extraction, common themes emerged that appeared in more than one study. A measure or outcome was considered a theme if it appeared in more than a single study [28]. If consistent findings appeared in more than four studies, this was deemed strong evidence for an effect. If consistent findings appeared in two of two, or two of three studies, then this was considered suggestive of an effect. However, given the heterogeneity of study designs and small number of studies that met final eligibility for inclusion, it was not possible to conduct higher level quantitative methods (i.e., meta-analysis) to coalesce the findings more broadly.

## 3. Results

### 3.1. Overview

Ten studies were identified as meeting criteria for inclusion (Table 1). Eight studies utilized the AIMS to assess athlete identity. Two utilized the Athlete Identity Questionnaire. Of these quantitative studies, eight involved a cross-sectional design, one included a case series and one involved a longitudinal design. Of the studies, six included subjects within the United States. One study included athletes from Korea, one study assessed athletes from the United Kingdom, one study researched athletes in Canada and another study originated from the Netherlands. For these studies, three out of the ten studies were deemed good studies and the remainder were of fair quality, based on the AXIS appraisal tool (Table 2). Six out of the ten studies did not justify their sample sizes. Accounting for or describing non-responders remained an issue for a sizable number of studies. Themes that arose from the studies include demographics such as gender and ethnicity, sports participation, injury and mental health.

### 3.2. Demographics

Three studies explored differences in demographics and athlete identity. Anderson utilized the Athletic Identity Questionnaire (AIQ) (Appendix C) as an outcome variable and Padaki utilized the Athlete Identity Measurement Scale (AIMS) (Appendix B). In Anderson’s study of general elementary and middle school students (did not specifically limit/focus on athletes), gender, ethnicity and socio-economic status were all found to significantly influence views of physical activity and athletic identity responses. Non-Hispanic white students in elementary school had a higher mean value on the AIQ as compared to Hispanics and non-Hispanic black students, with the exception of receiving encouragement from teachers and friends. The study found lower ratings on athlete appearance and competence in Hispanic girls and non-Hispanic black boys. In the 4th–5th grade cohort, white girls had significant higher Appearance, Competence and Importance ratings as compared to the non-white groups. The lowest self-ratings across these three subgroups were in non-Hispanic black boys and Hispanic girls. In the 7th–8th grade cohort, boys had higher Importance ratings than girls. Hispanic students rated physical activity as less important than non-Hispanic whites. Amongst younger students, ethnic differences of physical activity and athletic identity were found stratified by weight categories. Normal-weight Hispanic children rated themselves significantly less regarding athletic Appearance as compared to normal-weight white elementary students. In addition, obese black elementary students rated physical activity as less important than both their white and Hispanic counterparts. The study found significant differences amongst adolescents of different ethnicities, revealing that Hispanic adolescents had lower ratings on Athletic Appearance than blacks and lower ratings on the Competence subscales than both white and black adolescent students. In the older age group, Anderson found encouragement for physical activity highest amongst white parents and higher rates of sport team participation in the white students [29]. Athletic Identity was also studied in youth experiencing injury. When assessing the effects of athletic identity in the setting of ACL injury and diagnosis, Padaki found no significant differences in levels of athletic identity amongst 24 male and females (50% female) or between younger and older patients [30]. Similarly, Pot’s study of 304 athletes found that AIMS scores were not different between genders or age [31]. In contrast, a sample of 63 (100% female) gymnasts were surveyed and asked to complete questions retrospectively for themselves at age 10 years, 15 years and then again for their current age. The researchers found athletic identity significantly increased between the ages of 10 and 15 years and then plateau (no significant difference between 15 years of age and current age) [32]. These studies were rated as fair to good quality as far as their bias risk.

### 3.3. Sports and Physical Activity Participation

Half of the studies included in the review investigated the effects of athletic identity on sports participation or physical activity levels. In a study by Anderson et al., athletic identity was examined for association with concurrent seven-day physical activity levels and sports team participation. Athletic identity positively correlated to seven-day physical activity levels and sports team participation in both elementary school and middle school students [33]. The components of the AIQ all worked synergistically in contributing to higher activity levels and organized sports participation. While gender and race were found to influence participation in sports and activity levels, athletic identity levels accounted for just as much variance between groups.

Two studies suggest that the concept of athlete identity can peak and then plateau for individuals along their continuum of participation in a sport. Researchers studied 168 male elite youth English footballers aged 16–18 years and assessed athletic identity in comparison against playing level, living arrangements and year of apprenticeship. There were no significant differences in AIMS scores when comparing across the different levels of competition. The mean social identity within AIMS was found to be significantly different in those athletes in their first year of apprenticeship as compared to those in the second year, with the first year athletes having a higher degree of viewing himself as occupying the role of “athlete.” [34]. As previously detailed, Houle found athletic identity to significantly increase between the ages of 10 and 15 years and then plateau [32]. Both studies were rated as fair quality. Nevertheless, the data seems to support a theory that youth adopt a stronger sense of identity as an athlete during their adolescent development years as compared to later in their training or participation.

Two studies assessed the effects of sports participation duration on athletic identity. One study looked prospectively at 304 elementary school children (48% female) in the Netherlands to determine whether participation in a weekly school European football program over 1 year would affect sport and student identity. Survey instruments included a modified AIMS and a Psychological Sense of School Membership tool. Those who participated during the entire competition season had higher levels of athlete identity at baseline compared to those not participating or those who dropped out. Regression analysis showed that participation in the football program did not influence athlete or student identity in a statistically significant manner, thus discrediting the authors’ socialization hypothesis [31]. The authors suggested that perhaps sporting activities outside of this organized school setting contribute more to an athlete’s sense of athletic identity. Another study assessed adolescents with mobility impairments to determine sport participation patterns as well as the relationship between athletic identity and actual/desired sport participation. The authors found that within the group of 47 adolescents (28% female), AIMS scores were higher in those who participated in more hours of sport per week as well as those who preferred to spend more time in sport [35]. All studies mentioned above were rated as fair in risk bias.

### 3.4. Injury

McKay’s study looked at psychosocial risk factors for injury risk within 316 elite youth hockey players in Calgary, Canada. Athletes were surveyed using the 7-item AIMS tool. The researchers found that the male athletes who scored under the 25th percentile for athletic identity had an odds ratio of 1.53 (CI, 1.05–2.22) for risk of first injury. Conversely, those athletes who scored above the 75th percentile had an odds ratio of 2.28 (CI, 1.01–6.04) for increased risk of subsequent injury, which was not affected by early return to play [36]. This study was rated as having a lower risk of bias. Other studies included in the systematic review had a background of injury, but did not explicitly assess any potential link between athletic identity level and risk of injury.

### 3.5. Mental Health

Three studies examined how athletic identity intersects with mental health outcomes. One case series sought to examine the relationship of posttraumatic stress disorder symptoms and athletic identity in patients who sustained an ACL injury. Amongst the 24 patients studied, a significant percentage experienced PTSD symptoms amidst their ACL tear diagnosis; 87.5% experienced avoidance and 83.3% experienced intrusion. The terms, avoidance and intrusion, represent major symptoms seen in patients who experience post-traumatic stress disorder (PTSD) and are measured in this study using the Horowitz Impact of Event Scale-Revised (IES-R). Intrusion represents the inability to keep memories of an event from returning. Avoidance represents an attempt to avoid stimuli and triggers that may bring those memories back. Despite finding that older adolescents and females demonstrated significantly higher emotional disturbance scores, athletic identity scores were not found to significantly correlate with reported emotional trauma [30]. However, the study had a smaller sample size and may not be powered for that finding. Furthermore, the IES-R used to assess psychological trauma has not been established in the orthopedic literature.

Manuel aimed to examine factors that can affect an adolescent’s psychological state and outcomes during injury and recovery. The study tracked 48 adolescents (58% female) seen for an injury within an orthopedic surgery clinic over three months. The researchers used the original 10-item AIMS tool and found that those youth with higher athletic identity/exclusivity scores had higher Beck Depression Scale scores during their injury after controlling for injury severity and gender. Those depression scores decreased 6 and 12 weeks after the onset of injury [37].

Another project looked specifically at factors that could affect high school athletes in Korea’s physical education high schools. Within the 346 youth athlete cohort (32% female), athletic identity was found to have a positive correlation with athlete satisfaction, which in turn negatively correlated with stress and burnout. Stress was not found to have any correlation with athletic identity. The researchers found burnout to be a mediator between stress and athletic identity [38].

## 4. Limitations

There were some notable limitations in the body of research that assessed athletic identity in the youth population. In our body of studies, no two studies addressed the same research question, thus weakening any potential examination of associations. Moreover, the assessment of the quality of the research pointed to considerable levels of bias. The risk of bias table shows that many studies were weakly powered, failed to address internal consistency and negated to account for non-responders (Table 2).

We limited this review by age and there were some publications that included cohorts including the relevant topic but lacked median/mean athlete identity outcome scores, did not report ages of participants or the mean age of participants were outside of the range. However, our specific focus was on a younger population. In addition, we broadly looked at studies that included different scales of athletic identity, to include the AIMS and the AIQ. Future research on the scales and factor structure within the measurement tools should be performed and contemplated depending on the aim of the study and the target population.

Our review focused on quantitative studies that included a measurable scale of athletic identity. We chose to exclude qualitative studies to enable consistency of comparison, which may have limited the themes that arose in analysis, such as more nuanced intersections of athletic identity and gender or ethnicity. Other researchers have performed studies to assess narratives of athletes surrounding gender and age, focusing on the effects of athletic identity on sport culture or identity construction [39,40] to include more nuanced discourses around concepts of masculinity, femininity, motherhood and identity roles. For younger populations, that could translate into assessing the intersectionality of gender identity, ethnicity and athletic identity and how an individual navigates those junctions. Qualitative research can provide a rich description of those nuances and further ethnographic study involving younger athlete populations is needed.

## 5. Discussion

The aim of this systematic review was to amalgamate the current knowledge base regarding athletic identity amongst youth populations. Prior quantitative studies have examined the link between the level of athletic identity and other outcome measures such as adaptation to sports retirement [18,41], eating disorders [42], illicit behavior [9,43,44], aggressiveness [45] and injury [36]. Prior research has found amongst older athlete populations that those with strong athletic identity tend to have increased motivation or higher commitment to one’s sport [9]. Those with a more singular self-concept driven by athletic identity have been found to have more challenges with career transition [38]. Prior research has elucidated how exceptional demands of high level or elite sport can prevent athletes from engaging in a wide range of opportunities that support the development of a more mature and well-rounded individual identity. However, the question arises whether those effects occur earlier in one’s lifespan.

This review found 10 studies that involved the youth population and included a scale to measure athlete identity. There were insufficient studies to be able to make clear associations but there was some cross-study evidence that in adolescence, the transition to an initial higher level of play can also be correlated with a stronger sense of athletic identity [32,34]. In addition, those who participated during the entire competition season had higher levels of athlete identity at baseline compared to those not participating or those who dropped out, supporting a selection hypothesis of participation [34]. Several studies revealed the effect athletic identity can have on the mental health of youth, including depression and stress [30,37,38].

Athletes represent a unique population; involvement in organized sports places that individual within a complex social network that intersects with health, social-emotional development and societal expectations for entertainment [46]. Research has found that adolescents and young adults integrated in such a network may be less susceptible to psychosocial implications of anomie, a term coined by Durkheim to describe a breakdown of social norms that can leave an individual at risk for social isolation and mental health illness [47]. Prior studies have shown that sports participation can decrease one’s risk of hopelessness, depression and suicide ideation in college and high school and that intensity or frequency of involvement can also moderate those effects [48,49]; more research is needed to evaluate how early those effects take hold on a particular individual and what other psycho-social supports are needed to help a youth athlete engage in their sport in a healthy and sustainable way. Some studies suggest that excessive sports participation can negate the positive effects of athletic engagement [50]. For youth and adolescents, more studies are needed to assess whether that arises from a dose-response or if those effects instead rest on how an individual conceptualizes and assigns meaning to a particular sports experience, thus framing one’s athletic identity.

As youth are reengaging in the sports landscape amidst policy and funding changes arising from a pandemic that created structural, financial and staffing constraints [51], youth sport organizations, recreation leagues and schools need to consider how the intersection of identity and sports participation, or lack thereof, can influence health, psychosocial and longitudinal outcomes. In addition, assessing how a youth athlete may be affected by individual or team play and the interconnectedness of that social interaction in relation with athletic identity can frame the organization of youth development models in sport as well as recreation or competitive leagues. From there, targeted approaches to optimizing one’s objective and subjective experience in sports participation can possibly help with injury reduction, engagement with the sport and with others and mental health support.

As athletes interact with a diverse network of players that may influence their objective and subjective experience in play, another area of interest that merits more formal exploration involves the interplay between a youth athlete and a parent or coach. Regarding sport, parents serve as primary agents responsible for initiating their children’s participation in physical activity and sport and maintain a strong role in supporting those sport experiences [52]. Aspirations to obtain college scholarships or ascend the ranks to professional status exist within youth and adolescent athletes, but research has underscored the influence of parental motivation on their children [53]. These trends highlight a pervasive culture of elite, high-performing sport in the United States and the shift to externally regulated participation versus intrinsic incentivization. These trends in relation to athletic identity levels deserves more direct investigation.

## 6. Conclusions

The field of sports medicine would benefit from robust quantitative research that assesses the effects of athletic identity on specialization in sports and levels of burnout, stress and ego resiliency. Amidst a time when more youth athletes are participating in high level sport competition [54], the question arises as to whether an over-emphasis on performance, excellence and results affects youth athletes’ identity development, or particular levels of athletic identity in youth affect risk of burnout. As the youth sports landscape has transformed into a multi-billion dollar industry [55], more discussion and analysis is needed to assess the effects of that environment on youth sport as a vehicle for social identity and on a youth development model. During adolescence, a time when one’s identity is constructed based on various social interactions, a strong exclusivity in athletic identity can hamper the development of other elements of one’s individual identity. In addition, long-term outcomes of such research are also critical to explore, thus pointing to the need for more longitudinal research in this field. Further research should explore the concept of athletic identity saliency as one moves through an athletic career or training program and how thoughts of perceived success, professional progression, recruitment prospect or injury affect levels of athletic identity. Youth athletes can differ from adults, particularly with regards to identity formation and more research is needed if health care professionals and coaches are to optimally assist and support this population as they navigate through various sport participation trajectories amidst their growth and development and create tailored programs to support them.

## Figures and Tables

**Table 1:** Characteristics, Data Extraction from Articles.

1st Author	Journal	Year	Level of Evidence	Study Design	Study Participants (n)	Age	% Female	% Non-White	Athlete Identity Scale Used	Athlete Identity Scale Measurement Results	Other Outcomes	Main Findings	Quality
Anderson	American Journal Of Preventive Medicine	2009	IV	Cross-Sectional	391	Range 8–13 (mean 9.9) years	53	46	AIQ	Subscales used Appearance * 3.41 ± 0.69; Competence * 4.37 ± 0.57; Importance 4.07 ± 0.71; Encouragement from parents * 3.80 ± 0.85; Encouragement from friends 3.16 ± 1.07; Encouragement from teachers 3.31 ± 1.07	PAQ-C	AI is independently and positively associated with MVPA ^6^ and team participation. Competence and parental encouragement are significant correlates of physical activity.	Fair
Anderson	American Journal Of Preventive Medicine	2009	IV	Cross-Sectional	948	Range 11–15 (mean 13.6) years	61	54	AIQ	Subscales used Appearance 3.29 ± 0.72; Competence 4.23 ± 0.70; Importance * 3.82 ± 0.82; Encouragement from parents 3.83 ± 0.91; Encouragement from friends 3.23 ± 1.04; Encouragement from teachers 3.49 ± 1.14	MAQ-A	AI is independently and positively associated with MVPA6 and team participation. Sports team participation more positively affects athletic identity in adolescents than 7-day physical activity.	Fair
Anderson	Journal of Physical Activity and Health	2011	IV	Cross-Sectional	1830	Mean 10.1 years (Grades 4 & 5); 13.7 years (Grades 7 & 8)	N/A **	64	AIQ	Subscales used separated by ethnicity Appearance; Competence; Importance; Encouragement from parents; Encouragement from friends; Encouragement from teachers	BMI category	Gender, ethnicity and SES all significantly influenced AI responses. Non-Hispanic whites were significantly less overweight and obese and participated more in organized sports as compared to the Hispanics and non-Hispanic blacks and had higher mean value on the AIQ.	Fair
Manuel	Adolescent Health Brief	2002	IV	Cross-Sectional	48	Mean 17 years	58	85	AIMS	Total score 47.20 ± 9.78	Injury Severity Scale; Adolescent Cope Scale; Beck Depression Scale	AI/exclusivity scores were positively correlated with higher depressive symptoms after controlling for injury severity and gender.	Fair
Lee ^1^	Psychological Reports	2017	IV	Cross-Sectional	332	Mean 17.57 years	32.2	100	AIMS	Subscale scores Social ID: 3.81; Exclusivity: 3.55; Negative Affectivity: 3.82	Athletic Burnout; Athletic Stress; Athlete Satisfaction; Ego Resilience	AI was found to have a positive correlation with athlete satisfaction. Athlete satisfaction negatively correlated with stress and burnout. Stress was not found to have any correlation with AI. Burnout was found to be a mediator between stress AI.	Fair
Mitchell	Journal of Sports Sciences	2014	IV	Cross-Sectional	168	Range 16–18 years	0	N/A	AIMS	Total and Subscale scores Total: 40.5; Social ID: 16.4; Exclusivity: 11.5; Neg Affectivity: 12.3		There were not any significant differences AI level when comparing across different leagues. The mean social identity within AIMS was found to be significantly different in those athletes in their first year of apprenticeship as compared to those in the second year.	Fair
McKay	Clinical Journal Sports Medicine	2013	IV	Cross-Sectional	316	Mean 15 years	0	N/A	AIMS	Total score 55.62	Body Checking; Competitive State Anxiety Inventory-2R; Injuries	Athletes who scored low for AI (under the 25th percentile) had a 1.54 higher risk of first injury. Those athletes who scored high on the Athletic Identity Scale (above the 75th percentile) has a 2.28 increased risk of subsequent injury, which was not affected by early return to play.	Good
Houle ^2^	Journal of Sport Behavior—Study 1	2010	IV	Cross-Sectional	63	Range 18–22 years	100	6	AIMS	Total score at age 10: 34.67; at age 15: 37.82; at age current age: 37.41		AI significantly increased between the ages of 10 and 15 years and then plateaued. Former athletes had significantly lower current levels of AI as compared to current athletes. Non-athletes had overall lower levels of AI for all measurement times as compared to current and former athletes.	Fair
Houle ^3^	Journal of Sport Behavior—Study 2	2010	IV	Cross-Sectional	179	Range 19–24 years	N/A	17	AIMS	Total score across age time points (age 10, age 15, current age), separated according to athletic status			Fair
Padaki ^4^	Orthopaedic Journal of Sports Medicine	2018	IV	Case Series	24	Mean 14.5 ± 2.7 years	50	N/A	AIMS	Total score older age cohort: 54.1; younger age cohort: 54.5; Females: 56.6; Males: 53.4	PTSD via Horowitz impact of event scale, sport specialization	A significant percentage of patients experienced PTSD symptoms with diagnosis of an ACL tear. AI scores did not significantly correlate with reported emotional trauma. AI was similar among males and females as well as younger and older patients.	Good
Piatt	Disability and Health Journal	2018	IV	Cross-Sectional	47	Mean 15.57 ± 1.66 years	27.7	N/A	AIMS	Total score Males: 37.35; Females: 39.72; Middle School cohort: 37.6; High School cohort: 39.47; Higher hours for sports practice: 43.57; low time for sport practice: 36.36 *	Time spent in sports participation, preferred amount of time in sports participation	AIMS scores were higher in those who participated in more hours of sport per week as well as those who preferred to spend more time in sport each week. No statistically significant differences in AI based on gender or academic level.	Good
Pot ^5^	European Journal of Sport Science	2014	II	Prospective Longitudinal Cohort	304	Range 10–12 years	47	N/A	AIMS	Average scores separated by gender and intervention	School sports participation intervention, student identity- psychological sense of school membership scale, physical activity—Moderate to Vigorous Physical Activity Measure	Regression analysis showed participation in the school football program did not influence sport or student identity in the youth cohort.	Fair

AI (Athletic Identity); AIQ (Athlete Identity Questionnaire); AIMS (Athletic Identity Measurement Scale); PAQ-C (Physical Activity Questionnaire for Children); MAQ-A (Modifiable Activity Questionnaire for Adolescents). * Statistically significant on regression models. ** N/A: That particular study did not obtain/not measure that variable. ^1^ AIMS modified to 5 point scale. ^2^ Intercollegiate Gymnasts, Retrospective recall of subjects. ^3^ Self report non-athlete, former athlete, current athlete designation (former athlete N = 112; non-athlete N = 34; current athlete N = 33). ^4^ No average Athletic Identity reported, just differences in age, gender. ^5^ AIMS 5 item questionnaire adapted. ^6^ MVPA: Moderate to vigorous physical activity.

**Table 2:** AXIS Appraisal of Studies.

Author/Year	Anderson 2009	Anderson 2011	Manuel 2002	Lee 2017	Mitchell 2012	McKay 2013	Houle 2010	Padaki 2018	Piatt 2016	Pot 2014
Question										
Were the aims/objectives of the study clear?	**●**	**●**	**●**	**●**	**●**	**●**	**●**	**●**	**●**	**●**
Was the study design appropriate for the stated aim(s)?	**●**	**●**	**●**	**●**	**●**	**●**	**●**	**●**	**●**	**●**
Was the sample size justified?	**●**	**●**	**●**	**●**	**●**	**●**	**●**	**●**	**●**	**●**
Was the target/reference population clearly defined? (Is it clear who the research was about?)	**●**	**●**	**●**	**●**	**●**	**●**	**●**	**●**	**●**	**●**
Was the sample frame taken from an appropriate population base so that it closely represented the target/reference population under investigation?	**●**	**●**	**●**	**●**	**●**	**●**	**●**	**●**	**●**	**●**
Was the selection process likely to select subjects/participants that were representative of the target/reference population under investigation?	**●**	**●**	**●**	**●**	**●**	**●**	**●**	**●**	**●**	**●**
Were measures undertaken to address and categorize non-responders?	**●**	**●**	**●**	**●**	**●**	**●**	**●**	**●**	**●**	**●**
Were the risk factor and outcome variables measured appropriately to the aims of the study?	**●**	**●**	**●**	**●**	**●**	**●**	**●**	**●**	**●**	**●**
Were the risk factor and outcome variables measured correctly using instruments/measurements that had been trailed, piloted or published previously?	**●**	**●**	**●**	**●**	**●**	**●**	**●**	**●**	**●**	**●**
Is it clear what was used to determined statistical significance and/or precision estimates? (e.g., *p*-values, confidence intervals)	**●**	**●**	**●**	**●**	**●**	**●**	**●**	**●**	**●**	**●**
Were the methods (including statistical methods) sufficiently described to enable them to be repeated?	**●**	**●**	**●**	**●**	**●**	**●**	**●**	**●**	**●**	**●**
Were the basic data adequately described?	**●**	**●**	**●**	**●**	**●**	**●**	**●**	**●**	**●**	**●**
Does the response rate raise concerns about non-bias?	**●**	**●**	**●**	**●**	**●**	**●**	**●**	**●**	**●**	**●**
If appropriate, was information about non-responders described?	**●**	**●**	**●**	**●**	**●**	**●**	**●**	**●**	**●**	**●**
Were the results internally consistent?	**●**	**●**	**●**	**●**	**●**	**●**	**●**	**●**	**●**	**●**
Were the results presented for all the analyses described in the methods?	**●**	**●**	**●**	**●**	**●**	**●**	**●**	**●**	**●**	**●**
Were the authors’ discussions and conclusions justified by the results?	**●**	**●**	**●**	**●**	**●**	**●**	**●**	**●**	**●**	**●**
Were the limitations of the study discussed?	**●**	**●**	**●**	**●**	**●**	**●**	**●**	**●**	**●**	**●**
Were there any funding sources or conflicts of interest that may affect the authors’ interpretation of the results?	**●**	**●**	**●**	**●**	**●**	**●**	**●**	**●**	**●**	**●**
Was ethical approval or consent of participants attained?	**●**	**●**	**●**	**●**	**●**	**●**	**●**	**●**	**●**	**●**

Green: strong/yes; Yellow: moderate/not specifically reported; Red: weak/no.

## Data Availability

This review was registered at PROSPERO International prospective register for systematic reviews (#168594) and can be found at: https://www.crd.york.ac.uk/prospero/display_record.php?RecordID=168594, accessed on 15 April 2021.

## Appendix A. Full Search Strategy

Athlete Identity:

1- Identity OR identities OR identif *
2- Athlete OR athletes OR athletic OR jock *
3- 1 and 2
4- (Athletic identity questionnaire) or (Athletic identity measurement scale) or aiq or aims
5- 3 OR 4
6- #5 Filters: English

## Appendix B. Athletic Identity Measurement Scale, AIMS

**Table A1:** Original Athlete Identity Measurement Scale Items.

1.	I consider myself an athlete.
2.	I have many goals related to sport.
3.	Most of my friends are athletes.
4.	Sport is the most important part of my life.
5.	I spend more time thinking about sport than anything else.
6.	I need to participate in sport to feel good about myself.
7.	Other people see me mainly as an athlete.
8.	I feel bad about myself when I do poorly in sport.
9.	Sport is the only important thing in my life.
10.	I would be very depressed if I were injured and could not compete in sport.

**Table A2:** Reconceptualized Athlete Identity Measurement Scale Items.

1.	I consider myself an athlete.
2.	I have many goals related to sport.
3.	Most of my friends are athletes.
4.	Sport is the most important part of my life.
5.	I spend more time thinking about sport than anything else.
8.	I feel bad about myself when I do poorly in sport.
10.	I would be very depressed if I were injured and could not compete in sport.

Factors: Social Identity; Exclusivity: Negative Affectivity. B.W. Brewer, A.E. Cornelius. Norms and factorial invariance of the Athletic Identity Measurement Scale. *Academic Athletic Journal* 2001, 16: 103–113.

## Appendix C

**Table A3:** Athlete Identity Questionnaire, AIQ.

ITEM	
	APPEARANCE
1.	I look athletic, like a person who exercises.
4.	My body looks in shape
7.	I look like a person who is physically fit.
9.	It’s obvious to others that I’m flabby and out of shape.
17.	I look healthy- not overweight or underweight.
	COMPETENCE
2.	I have the ability to participate in several types of physical activities.
5.	In most physical activities, I feel I can become skilled with enough effort and practice.
11.	I’m confident of my athletic skills.
14.	I have skill in several sports or physical activities.
15.	I can perform well in at least one type of physical activity.
18.	I know I can get better at sports, exercise, or physical activity.
	IMPORTANCE OF PHYSICAL ACTIVITY
3.	After illness or injury, I begin exercising/doing physical activity as soon as possible.
6.	I’d be very upset if something prevented sports/exercise planned for me.
8.	I don’t let things get in the way of my exercise/sports/physical activities.
10.	I love to exercise.
12.	I’d rather spend time playing sports or being active than sitting around or watching TV.
13.	I love to play active sports.
16.	I put a lot of effort into sports or exercise.
19.	I really like to be physically active.
	ENCOURAGEMENT FROM OTHERS
	Parents: I have parents/family who: // Best friends: My best friends: Teacher or other adult: I have a teacher or another adult outside my family who:
20., 27., 34.	encourage(s) me to exercise or be physically active.
21., 28., 35.	exercise(s) or work(s) out along with me.
22., 29., 36.	give(s) me words of confidence concerning sports or exercise.
23., 30., 37.	watch(es) me closely and give(s) me feedback on what I’m doing.
24., 31., 38.	have/has spent time teaching me how to play a sport or do a physical activity.
25., 32., 39.	are/is proud of me when I exercise.
26., 33., 40	are/is willing to help me in every way when it comes to sports or exercise.

Response anchors: 1. Strongly disagree; 2. Somewhat disagree; 3. Neither agree nor disagree; 4. Somewhat agree; 5. Strongly agree. Source: Anderson, C.B., L. Masse, C. Hergenroeder and C. Albert. Factorial and Construct Validity of the Athletic Identity Questionnaire for Adolescents. *Med Science in Sports & Exercise* 2007; 39(2): 59–69.
